# X-box Binding Protein 1 is a Potential Immunotherapy Target in Ovarian Cancer

**DOI:** 10.3389/fgene.2022.818917

**Published:** 2022-08-04

**Authors:** Yanhui Jiang, Lewei Yang, Ling Jiang, Wenyan Yu, Zhongwen Jin, Yeqing Qiu, Yifeng Liao, Jihong Liu, Hongyu Zhang

**Affiliations:** ^1^ Cancer Center, The Fifth Affiliated Hospital of Sun Yat-Sen University, Zhuhai, China; ^2^ Department of Pathology, Zhuhai Hospital of Integrated Traditional Chinese and Western Medicine, Zhuhai, China; ^3^ Department of Gynecology, The Fifth Affiliated Hospital of Sun Yat-Sen University, Zhuhai, China; ^4^ Department of Gynecologic Oncology, State Key Laboratory of Oncology in South China, Sun Yat-Sen University Cancer Center, Guangzhou, China

**Keywords:** XBP1, immune checkpoint, immunogenic cell death, genomic alteration, bioinformatics analysis, ovarian cancer

## Abstract

The allure of potentially dramatic and durable responses to immunotherapy has driven the study of several immune checkpoint inhibitor (ICI) agents in ovarian cancer. However, the results of ICI therapy in ovarian cancer have been rather disappointing. It is important to understand the reasons for the poor efficacy of ICI in ovarian cancer and to look for new targets for immunotherapy. To solve this problem, ovarian cancer–associated datasets were individually collected from The Cancer Genome Atlas (TCGA)、International Cancer Genome Consortium (ICGC)、Genotype-Tissue Expression (GTEx), and comprehensively performed to expression, prognostic, pathological correlation, genomic and immunologic analyses of reported all immune checkpoints by Gene Expression Profiling Interactive Analysis 2 (GEPIA2), Tumor and Immune System Interaction Database (TISIDB), cBio Cancer Genomics Portal (cBioPortal), and Kaplan-Meier Plotter. We concluded that those well-identified immune checkpoints might not be ideal targets for ovarian cancer immunotherapy. Intriguingly, the genomic alteration of X-box binding protein 1 (XBP1), the important mediator of chemotherapy-induced cancer immunogenic cell death, was found to be a potential coregulator of immune checkpoints in ovarian cancer. Importantly, XBP1 was detected to be highly expressed in ovarian cancer compared with normal ovarian tissue, and high XBP1 expression significantly benefits both overall survival (OS) and disease-free survival (DFS) of ovarian cancer patients. More importantly, XBP1 was further observed to be closely related to anti-tumor immunity in ovarian cancer, including multiple T-cell signatures and immunity-killing molecules. In conclusion, upregulating XBP1 rather than targeting immune checkpoints represents a potentially more efficient approach for ovarian cancer therapy.

## Introduction

Ovarian cancer (OC) has been reported to be the fifth leading cause of death among females, and an estimated 21,410 new cancer cases and 13,770 cancer-related deaths occurred in the United States in 2020 (Siegel et al., 2021). Standard treatment, including surgery and chemotherapy, is usually effective at inducing remission, but in 70–80% of patients, the cancer recurs within 2 years (Matulonis et al., 2016). Despite numerous efforts to improve the efficacy of surgery, chemoradiotherapy, and targeted treatments, few notably better therapeutic strategies for treating ovarian cancer in daily clinical practice have been discovered in recent years (Jayson et al., 2014). Recently, immune-checkpoint inhibitor (ICI) targeting programmed death-1 (PD-1) and programmed death ligand-1 [PD-L1 (CD274)] have been investigated in ovarian cancer, but the clinical response of these inhibitors is quite limited (Disis et al., 2019; Matulonis et al., 2019). Additionally, multiple immune checkpoints have been reported, including PD-L2 (PDCD1LG2), CTLA-4, LAG3, TIM-3, HHLA2, CD80, CD200, CD112 (NECTIN2), VSIR(VISTA), TNFRSF14, PVR, CD86, LGALS9, ICOSLG, TNFSF9, CD48, TNFSF18, VTCN1, TNFSF4, and CD70 (Pardoll, 2012; Wei et al., 2018). However, whether and how those immune checkpoints are involved in the expression, prognosis, pathological correlation and the therapeutic efficacy of ovarian cancer remain largely unknown. Thus, it is imperative to understand the underlying the clinical significance of immune checkpoints and to identify novel immunotherapy target in ovarian cancer.

Chemotherapy is an important treatment method for ovarian cancer. It can not only induce apoptosis, but also immunogenic cell death (ICD) of tumor cells. Once tumor cells undergo ICD, dying cells release damage-related molecular patterns (DAMPs), which then recruit antigen-presenting cells to engulf and process tumor cell antigens, and further activate adaptive immune response (Michaud et al., 2011). Therefore, mediating ICD key factors play an important role in activating the body’s anti-tumor immunity and producing long-term anti-tumor effects.

Immunogenic X-box binding protein 1 (XBP1) is an important factor mediating immunogenic cell death (ICD) (Schardt et al., 2009). It is a unique basic-region leucine zipper (bZIP) transcription factor whose dynamic form is controlled by an alternative splicing response upon disturbance of homeostasis in the endoplasmic reticulum (ER) and activation of the unfolded protein response (UPR) (Walter and Ron, 2011). Researches show that ICD is a kind of pro-inflammatory cell death, and the key to the occurrence of ICD is the production of persistent reactive oxygen species (ROS) and ER stress (Kepp et al., 2013; Jeong et al., 2021; Ma et al., 2022). XBP1 communicates with the foremost conserved signalling component of the UPR and is essential for cell fate determination in response to ER stress (ERS) in ICD process (Chen et al., 2020).

Intriguingly, Chemotherapy can activate endoplasmic reticulum stress to produce ICD by regulating the expression of intracellular XBP1 in multiple cancers (eg., melanoma, colorectal carcinoma, and acute myeloid leukemia) (Schardt et al., 2009; Pozzi et al., 2016; Prieto et al., 2019). Importantly, Glimcher et al. previously reported that XBP1 controls anti-tumor immunity by disrupting dendritic cell homeostasis in ovarian cancer (Cubillos-Ruiz et al., 2015). Moreover, Cubillos-Ruiz et al. found that IRE1α-XBP1 controls T cell function in ovarian cancer by regulating mitochondrial activity (Song et al., 2018). Thus, XBP1 might be a potential immunotherapy target.

With the advancement of genomic investigation technology, it has become an effective method to accelerate clinical and translational cancer research and treatment (Winters et al., 2018; Ben-David et al., 2019; Keenan et al., 2019). Recent researches have shown that immune checkpoints and some important gene-expression patterns are significantly correlated with the disease prognosis of several specific carcinomas, and can also be used to predict the efficacy of certain cancer types treated by cancer immunotherapy (Rizvi et al., 2015; Mandal et al., 2016; Luke et al., 2019). Using a high-throughput sequencing database, our results showed that XBP1 couples with immune checkpoints in ovarian cancer. Therefore, this study attempted to clarify the expression, prognosis, pathological correlation and therapeutic efficacy of ovarian cancer, and propose more suitable strategies for improving anti-ovarian cancer immunity.

## Materials and Methods

### Gene Expression Profiling Interactive Analysis 2 Database Analysis

Gene Expression Profiling Interactive Analysis 2 (GEPIA2, http://gepia2.cancer-pku.cn) (Tang et al., 2019), a web-based tool to deliver fast and customizable functionalities based on TCGA, ICGC, and GTEx data. It offers customizable functions such as tumor/normal differential expression analysis, profiling according to cancer types or pathological stages, patient survival analysis, similar gene detection, correlation analysis, and dimensionality reduction analysis. In this research, we used GEPIA2 to analyze the gene expression, pathological stages, correlation analysis and patient survival analysis in ovarian cancer. Differential analysis of gene expression was subjected to one-way ANOVA, and genes with higher |log2FC| values (>1) and lower q-values (<0.01) were defined as differentially expressed genes. Differential expression of log-scaling in different pathological stages was assessed by log2(TPM+1), and a Pr(>F)<0.05 was recognized as statistically significant. The Kaplan-Meier method with 50% cutoff for both low- and high-expression groups was used to analyze Overall survival (OS) and disease-free survival (DFS). Hypothesis test was assessed by Log-rank test. The statically significant difference was considered when a *p*-value is <0.05. Pair-wise gene expression correlations were analyzed using Spearman’s rank correlation coefficient, and a *p*-value<0.05 was recognized as statistically significant.

### cBioPortal Database Analysis

cBio Cancer Genomics Portal (cBioPortal) (http://cbioportal.org) provides a web resource for exploring, visualizing, and analyzing multidimensional cancer genomics data (Cerami et al., 2012). cBioPortal was used to conduct visualization and comparison of genetic alterations. Log2 odds ratio, *p*-value and q-value were used to assessed the co-occurrence and mutual exclusivity of genetic alterations between inquired gene and each immune checkpoint. Log2 odds ratio>0 was considered to be co-occurrence; log2 odds ratio<0 was recognized as mutual exclusivity. The statically significant difference was considered when a *p*-value or q-value is <0.05.

### Kaplan–Meier Plotter Database Analysis

The Kaplan-Meier plotter (Nagy et al., 2018) is able to assess the effect of 54 k genes on survival in 21 cancer types. The largest datasets include breast, ovarian, lung, and gastric cancer. In this study, it was used to evaluate the prognostic value of XBP1 mRNA expression in which ovarian cancer patients were split into high and low expression group based on median values of mRNA expression and validated by K-M survival curves, with the hazard ratio (HR) with 95% confidence intervals (CI) and log rank *p*-value. The statically significant difference was considered when a *p*-value is < 0.05.

### Tumor and Immune System Interaction Database Analysis

Tumor and Immune System Interaction Database (TISIDB, http://cis.hku.hk/TISIDB) is a user-friendly web portal, which contains a summary of 988 immune-related antitumor genes for 30 TCGA cancer types (Ru et al., 2019). The associations between gene expression and immune features, including lymphocytes, immunomodulators (immunoinhibitors, immunostimulator, and MHC molecule), subtypes, and chemokines, were calculated by high-throughput data analysis. In this research, we used the TISIDB web to analyze the correlations between XBP1 expression and lymphocytes, and subtype immunomodulators in ovarian cancer. The surveyed immunomodulators were extracted from Charoentong’s study (Ru et al., 2019), and each Spearman’s rank correlation between investigated gene and a unique immunomodulators in an individual cancer type was organized into the designated heatmap.

## Results

### Prognostic Analysis of Immune Checkpoints in Ovarian Cancer

**FIGURE 1:**
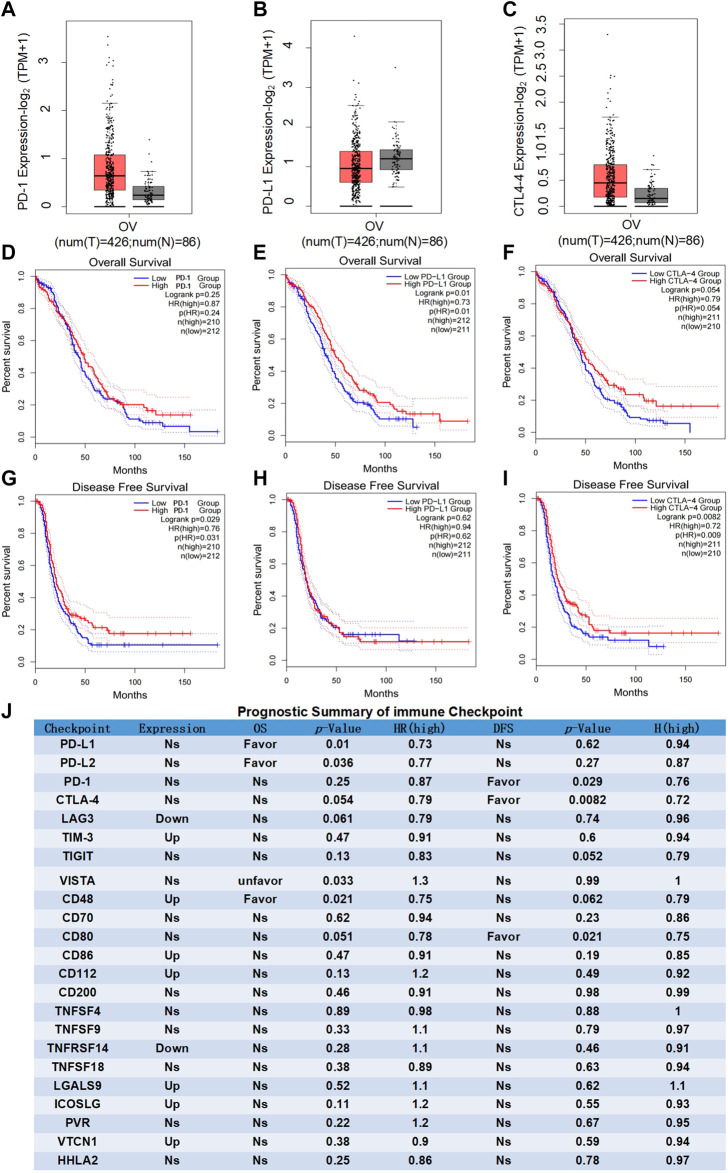
Prognostic analysis of immune checkpoints in ovarian cancer. **(A–C)** Differential expression analysis of common immune checkpoints in ovarian cancer. Box plots with jitter (size = 0.5) for comparing PD-1 **(A)**, PD-LI **(B)** and CTLA-4 **(C)** expression in ovarian cancer and normal ovarian tissues were made by GEPIA. T, tumor samples; N, normal samples. One-way ANOVA was used for differential analysis. Genes with higher |log2FC| values (>1) and lower q-values (<0.01) were recognized as differentially expressed genes. D-F, OS analysis of common immune checkpoints in ovarian cancer. Kaplan-Meier OS curves comparing the groups with different expression levels of PD-1 **(D)** PD-L1 **(E)** and CTLA-4 **(F)** in ovarian cancer (TCGA tumor) were made by GEPIA. G-I, DFS analysis of common immune checkpoints in ovarian cancer. Kaplan-Meier DFS curves comparing the groups with different expression levels of PD-1 **(G)**, PD-L1 **(H)** and CTLA-4 **(I)** in ovarian cancer (TCGA tumor) were made by GEPIA. Blue line: low-expression groups (50% cutoff); red line: high-expression groups (50% cutoff). Hypothesis test method is log-rank test. The Cox proportional hazard ratio and the 95% confidence interval information were also included in the survival plots. *p*-value < 0.05 was recognized as statistically significant. **(J)** Prognostic summary of the immune checkpoints in ovarian cancer. The detailed differential expression profile, OS, DFS, *p*-value, and HR (high) of all common immune checkpoints were individually summarized as indicated. The statically significant difference was considered when a *p*-value is <0.05. (Up: upregulated in tumor; Favor: favorable to survival; Unfavor: unfavorable to survival; Ns: nonsignificant).

Recent researches show that common immune checkpoint inhibitors such as PD-1/PD-L1 and CTLA-4 are only effective in patients with advanced/recurrent ovarian cancer, but the clinical efficacy is very limited. Differential expression analysis showed that the expression levels of PD-1/PD-L1 and CTLA-4 in ovarian cancer were insignificant compared with normal ovarian tissues (Figures 1A–C), and the expression in advanced ovarian cancer tissues was significantly lower than that in early ovarian cancer tissues (Supplymentary Figures S1A–C). Moreover, as BRCA1/BRCA2 mutations are confirmed to be important high-risk factors for the development of ovarian cancer. We further analyzed the correlation between PD-1/PD-L1/CTLA-4 and BRAC1/BRAC2. The results showed that the correlations of PD-1/PD-L/CTLA-4 and BRAC1 (Supplymentary Figures S1D–F), as well as PD-1/PD-L1/CTLA-4 and BRAC2 (Supplymentary Figures S1G–I), were actually nonsignificant in ovarian cancer. Furthermore, the survival analyses of patients with ovarian cancer clearly indicated that the expression levels of PD-1/PD-L1/CTLA-4 had no significant influence on overall survival (OS) (Figures 1D–F), and PD-1/CTLA-4 had significant influence on disease-free survival (DFS) while PD-L1 had nonsignificant (Figures 1G,I). These results indicated that the clinical significance of PD-1/PD-L1/CTLA-4 and clinical response of PD-1/PD-L1/CTLA-4 inhibitors are limited in ovarian cancer.

In addition to PD-1/PD-L1 and CTLA-4 as the most important immune checkpoints, emerging evidence indicates LAG3, TIM-3, TIGIT, VISTA, and other immune checkpoints are the representative ones in malignant tumor (Pardoll, 2012; Wei et al., 2018). Comprehensive analysis found that there were no advantage results in the prognostic analyses for any of the presentative immune checkpoints (Figure 1J). Summarizing the results of the comprehensive analysis can be divided into three types of conditions. (a) nonsignificant expression between ovarian cancer and normal ovarian tissues (eg, PD-1 and PD-L1); (b) upregulated or downregulated in ovarian cancer but no significant difference existed in the OS and DFS of ovarian cancer patients (eg, TIM-3 and LAG3). (c) statistically significant expression in ovarian cancer, closely related to the OS or DFS of ovarian cancer patients (favorable or unfavorable), but with both stimulatory and inhibitory potential on immune system (eg, CD48). More importantly, the relatively weak and even nonsignificant correlations were evaluated between immune checkpoints and pathological stage or BRAC1/BRAC2 expression, respectively (Supplymentary Figure S1J). These results suggested that immune checkpoints themselves might not be ideal immunotherapy targets for ovarian cancer.

### X-Box Binding Protein 1 is a Potential Coregulator of Immune Checkpoints in Ovarian Cancer

**FIGURE 2:**
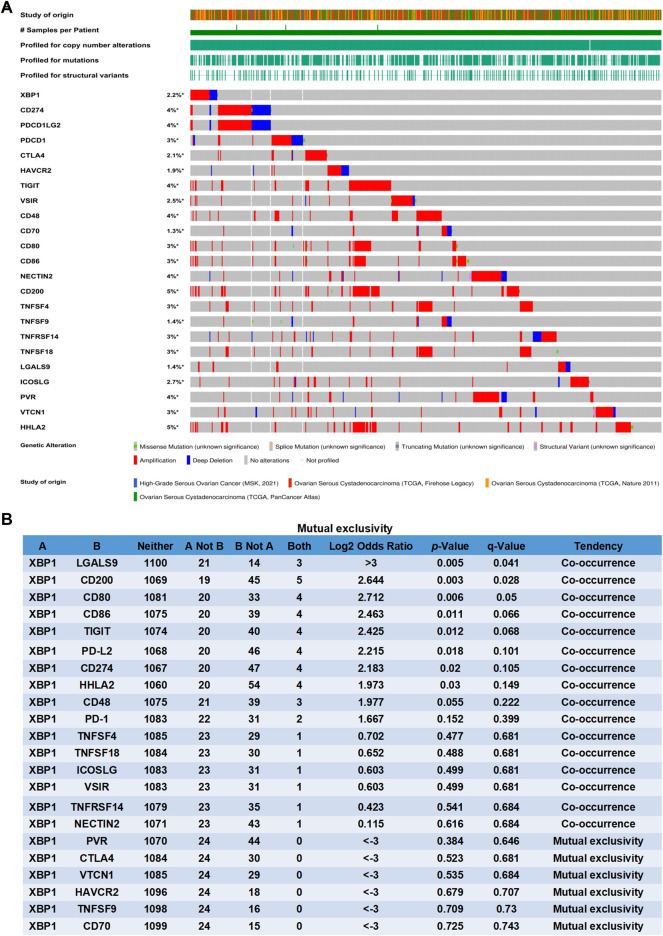
XBP1 is a potential coregulator of immune checkpoints in ovarian cancer. **(A)** Oncoprint of XBP1 and immune checkpoint alteration in ovarian cancer. Summative visualization of cases with multiple genetic alterations of XBP1 and immune checkpoints (originated from four studies) were individually shown by cBioPortal as indicated, including deep deletion, amplification, structural variant, truncating mutation, missense mutation, and splice mutation. **(B)** Mutual-exclusivity analysis between XBP1 and multiple-immune checkpoints in ovarian cancer. The altered relationship between XBP1 and each immune checkpoint, such as co-occurrence and mutual exclusivity, was presented as indicated. The detailed log2 odds ratio, *p*-value, q-value, and tendency were individually presented in each panel. log2 odds ratio>0 was considered to be Co-occurrence and log2 odds ratio<0 was considered to be Mutual exclusivity. The statically significant difference was considered when a *p*-value or q-value is <0.05.

Previous studies found that several key molecules have been identified that are involved in mediating immunogenic chemotherapy, including calreticulin (CALR), high-mobility group box 1 (HMGB1), protein disulfide isomerase family A member 3 (PDIA3), pannexin 1 (PANX1), annexin A1 (ANXA1), type I interferon receptor 1 (IFNAR1), and Immunogenic X-box binding protein 1 (XBP1) (Schardt et al., 2009; Ma et al., 2017; Galluzzi et al., 2020). Through the analysis of the cBioPortal database, we found that the genomic investigation revealed that XBP1 was actually involved in the alteration of immune checkpoints in ovarian cancer. The general landscape of XBP1 and immune checkpoint alteration in ovarian cancer was compactly visualized, including amplification, deep deletion, fusion, structural variant, truncating, missense, and splice mutations (Figure 2A). The detailed relationship between XBP1 and each representative immune checkpoint was individually presented as indicated in Figure 2B. When a *p*-value is <0.05, the XBP1 alteration showed co-occurrence rather than mutual exclusivity with extensive immune checkpoints, including LGALS9, CD200, CD80, CD86, TIGIT, PDCD1LG2, CD274, HHLA2. when a q-value is <0.05, the XBP1 alteration showed co-occurrence rather than mutual exclusivity with extensive immune checkpoints, including LGALS9, CD200, CD80. These results strongly indicate that XBP1 couples with immune checkpoints in ovarian cancer. Thus, it can be concluded XBP1 is a potential coregulator of immune checkpoints in ovarian cancer.

### Prognostic Analysis of X-Box Binding Protein 1 in Ovarian Cancer

**FIGURE 3:**
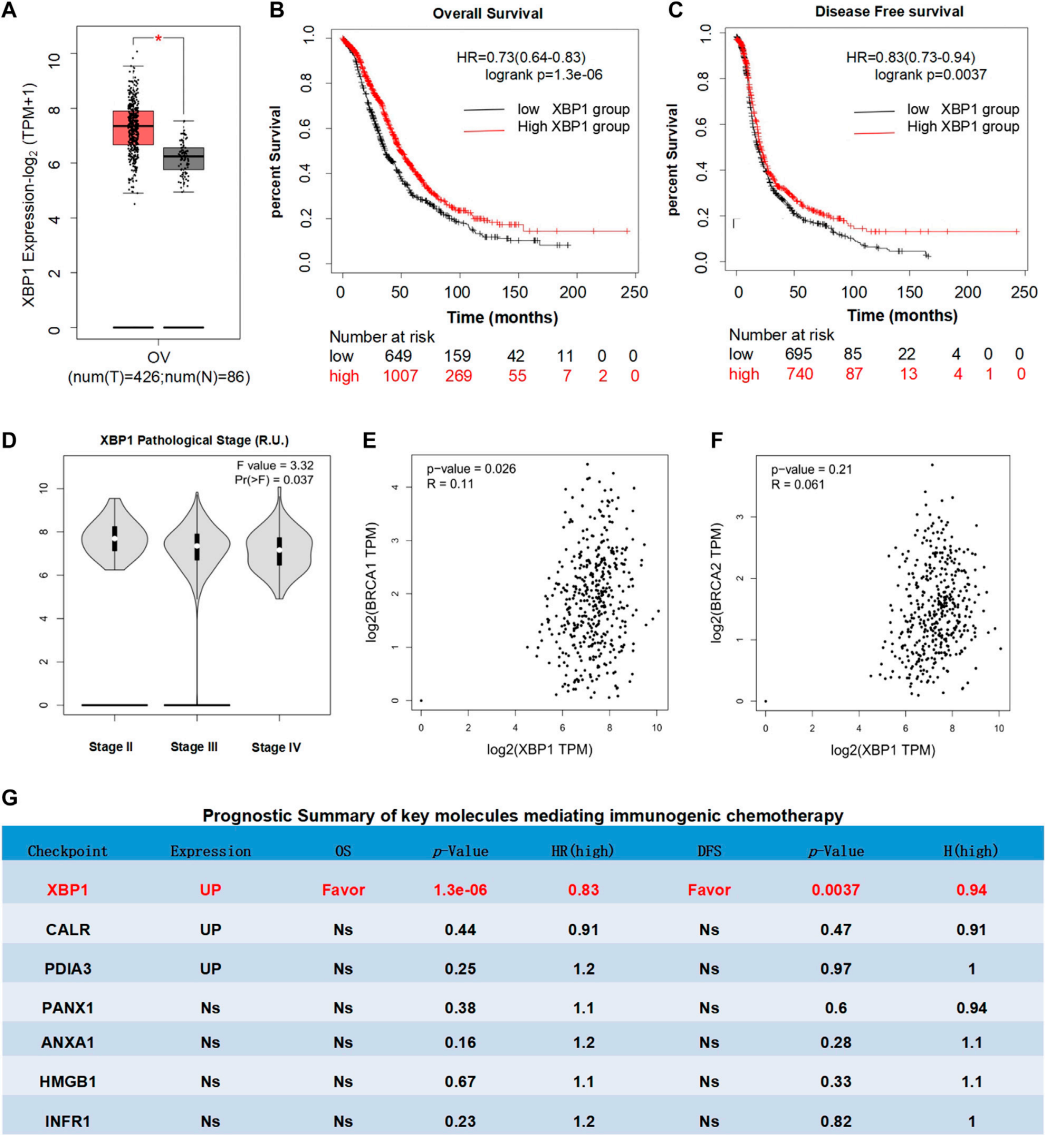
Prognostic analysis of XBP1 in ovarian cancer. **(A)** Differential expression analysis of XBP1 in ovarian cancer. Box plots with jitter (size = 0.5) for comparing XBP1 **(A)** expression in ovarian cancer and normal ovarian tissues were made by GEPIA. T, tumor samples; N, normal samples. One-way ANOVA was used for differential analysis. Genes with higher |log2FC| values (>1) and lower q-values (<0.01) were considered to be differentially expressed genes. **(B)** OS analysis of XBP1 in ovarian cancer. **(C)** DFS analysis of XBP1 in ovarian cancer. Kaplan-meier Plotter generates Kaplan-Meier OS **(B)** and DFS **(C)** curves comparing the groups with different expression levels of XBP1 in ovarian cancer. Blue line: low-expression group; red line: high-expression group. Log-rank test was used for hypothesis test. The Cox-proportional hazard ratio and the 95% confidence interval information were also included in the survival plots. The statically significant difference was considered when a *p*-value is <0.05. **(D)**. Different pathological stages analysis of XBP1 in ovarian cancer. Differential expression of log-scaling in different pathological stages was assessed by log2(TPM+1), and a Pr (>F) < 0.05 was considered to be statistically significant. **(E)** Correlation analysis of BRCA1 and BXP1 in ovarian cancer. **(F)** Correlation analysis of BRCA1 and BXP1 in ovarian cancer. The pair-wise gene expression correlations of two genes were made by GEPIA, or between one gene and BRCA1/BRCA2 in ovarian cancer (TCGA tumor) using Spearman method. The detailed *p*-value and R were individually presented as indicated in each panel.The statically significant difference was considered when a *p*-value is <0.05. **(G)** Prognostic Summary of key molecules mediating immunogenic chemotherapy. The detailed differential expression profile, OS, DFS, *p*-value, and HR (high) of key molecules mediating immunogenic chemotherapy were individually summarized as indicated. The statically significant difference was considered when a *p*-value is <0.05. (Up: upregulated in tumor; Favor: favorable to survival; Unfavor: unfavorable to survival; Ns: nonsignificant).

To investigate the therapeutic effect of XBP1-based therapy in ovarian cancer, the expression, prognostic and pathological correlation of XBP1 was further analyzed in detail. The results revealed that XBP1 is highly expressed in ovarian cancer in comparison to the normal control tissue (Figure 3A) and high XBP1 expression largely favored the OS and DFS of ovarian cancer patients (Figures 3B,C). In addition, the expression in advanced ovarian cancer tissues was significantly lower than that in early ovarian cancer tissues (Figure 3D). Since mutations in BRCA1 and BRCA2 were identified as major causes of ovarian cancer, the relationship between XBP1 and BRAC1/BRAC2 was individually analyzed. The results showed that the positive correlations of XBP1 and BRAC1 (Figure 3E), as well as XBP1 and BRAC2 (Figure 3F).

Meanwhile, the expression and prognosis of other representative regulatory factors of immunogenic chemotherapy was also analyzed as indicated in (Figure 3G). Firstly, the expression analyses of patients with ovarian cancer showed that the expression levels of CALR and PDIA3 in ovarian cancer were significantly up-regulated in compared with normal control tissues, while the expression of PANX1, ANXA1, HMGB1, and IFNAR1 were nonsignificant. Secondly, the survival analyses of patients with ovarian cancer clearly indicated that the expression levels of CALR, PDIA3, ANXA1, HMGB1, IFNAR1, and PANX1 had no significant influence on overall survival (OS) and disease-free survival (DFS). These results further confirmed that among the key mediators of immunogenic chemotherapy, XBP1 is potentially the only one favorable for the survival of ovarian cancer patients. Thus, XBP1 might be an ideal target for ovarian cancer.

Moreover, we further analyzed that the clinical significance of XBP1 in multiple human cancers, the expression profiles of XBP1 were observed across 33 major types of human cancer in The Cancer Genome Atlas (TCGA) database. Compared with paired normal tissues, XBP1 was expressed at higher levels in breast invasive carcinoma, colon adenocarcinoma, lymphoid neoplasm diffuses large B-cell lymphoma, glioblastoma multiforme, acute myeloid leukemia, brain lower grade glioma, ovarian cancer, rectum adenocarcinoma, testicular germ cell tumors, thymoma, uterine corpus endometrial carcinoma (Supplymentary Figure S2), suggesting XBP1 might be also a suitable target in these cancer types.

### Immune Correlation of X-Box Binding Protein 1 in Ovarian Cancer

**FIGURE 4:**
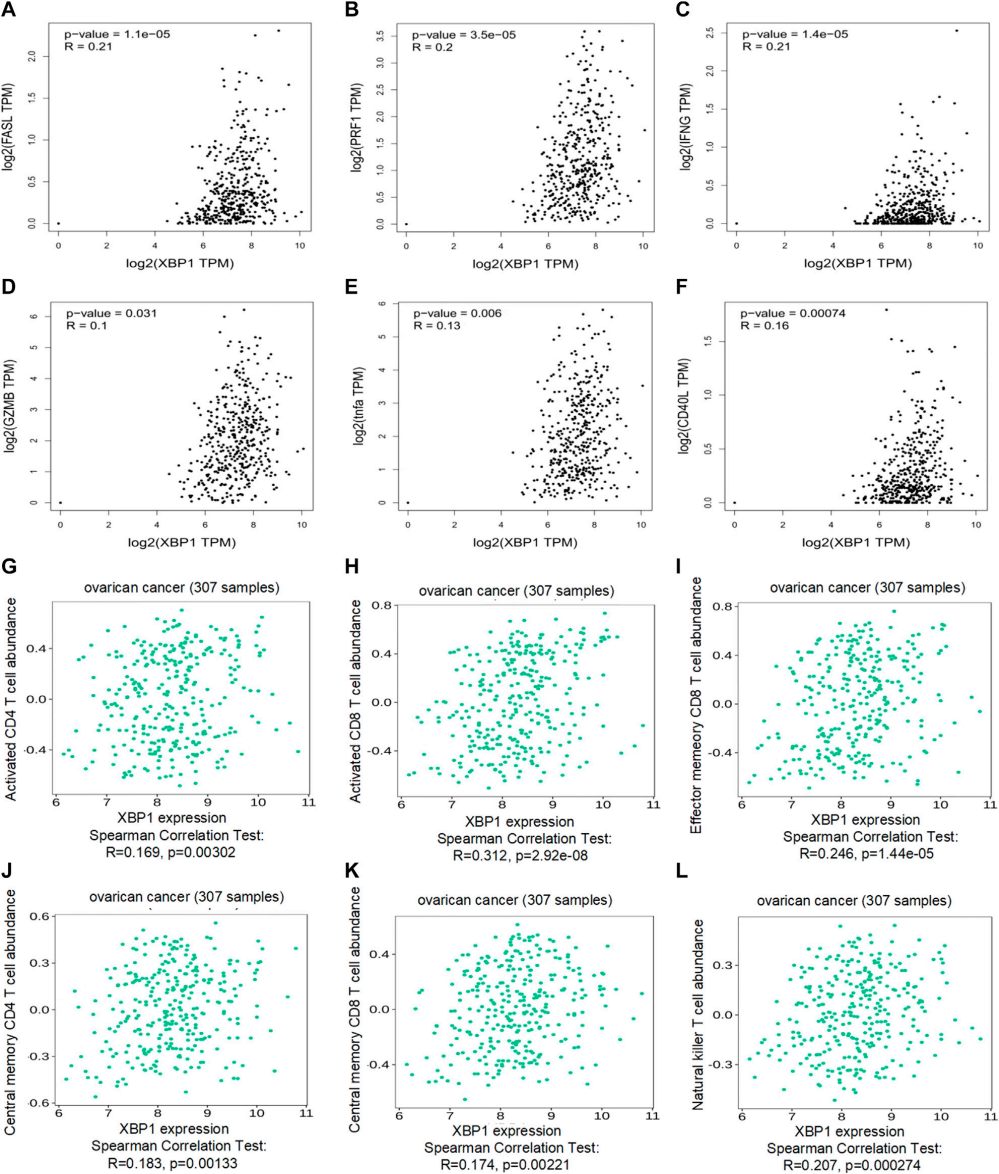
Immune correlation of XBP1 in ovarian cancer. **(A)** Correlation analysis of XBP1 and FASL in ovarian cancer. **(B)** Correlation analysis of XBP1 and RPF1 in ovarian cancer. **(C)** Correlation analysis of XBP1 and IFNG in ovarian cancer. **(D)** Correlation analysis of XBP1 and GZMB in ovarian cancer. **(H)** Correlation analysis of XBP1 and TNFa in ovarian cancer. **(E)** Correlation analysis of XBP1 and CD40L in ovarian cancer. **(F)** Correlation analysis of XBP1 and actived CD4 T cell Abundance in ovarian cancer. **(G)** Correlation analysis of XBP1 and actived CD4 T cell Abundance in ovarian cancer. **(H)** Correlation analysis of XBP1 and actived CD8 T cell Abundance in ovarian cancer. **(I)** Correlation analysis of XBP1 and effector memeory CD4 T-cell Abundance in ovarian cancer. **(J)** Correlation analysis of XBP1 and central memory CD4 T-cell Abundance in ovarian cancer. **(K)** Correlation analysis of XBP1 and central memory CD8 T-cell Abundance in ovarian cancer. **(L)** Correlation analysis of XBP1 and Nature killer T-cell Abundance in ovarian cancer. The pair-wise gene expression correlations of two genes were made by GEPIA, or between one gene and several signatures in ovarian cancer (TCGA tumor) using Spearman method. The detailed *p*-value and R were individually presented as indicated in each panel. The statically significant difference was considered when a *p*-value is <0.05.

To explore the therapeutic potential of XBP1-based immunotherapy in ovarian cancer, the immuno-logic correlation of XBP1 was further analyzed in detail. We found that XBP1 was significantly correlated with key immunity-killing molecules, including FASL (Figure 4A), PRF1 (Figure 4B), IFNG (Figure 4C), GZMB (Figure 4D), TNFa (Figure 4E), and CD40L (Figure 4F) in ovarian cancer. Moreover, Analysis of the correlation between the expression of XBP1 and immune signatures revealed that the expression of XBP1 was positively correlated with the abundance of major immune killer cells, including activated CD4 T cell (Figure 4G), activated CD8 T cell (Figure 4H), effector memeory CD8 T cell (Figure 3I), central memory CD4 T cell (Figure 4J), central memory CD8 T cell (Figure 4K), Natural killer T cell (Figure 4L). Together, this evidence strongly suggests that XBP1 had a significant impact on the antitumor immune response in ovarian cancer therapy.

**FIGURE 5:**
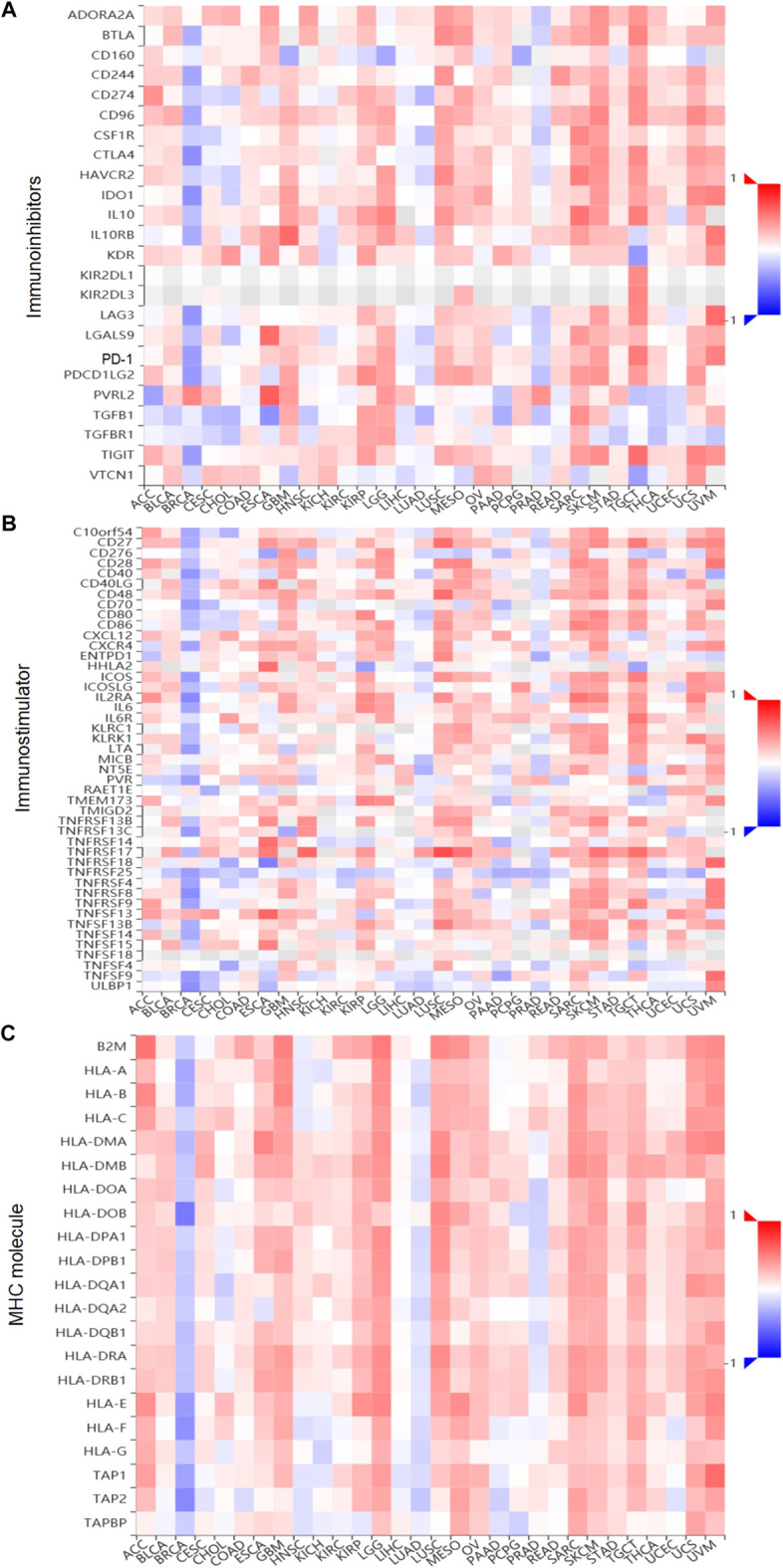
(Continued).

Importantly, to deeply comprehend the association between expression of XBP1 and immune regulation, relations between three kinds of immunomodulators and XBP1 expression were further analyzed across 30 cancer types. The results showed that XBP1 expression levels correlated positively with the relative abundance of major immunoinhibitor/immunostimulator in several specific cancers, such as adrenocortical carcinoma, bladder urothelial carcinoma, esophageal carcinoma, glioblastoma multiforme, kidney renal papillary cell carcinoma, acute myeloid leukemia, lung squamous cell carcinoma, ovarian cancer, sarcoma, skin cutaneous melanoma, testicular germ cell tumors, uterine carcinosarcoma, and uveal melanoma (Figures 5A,B), while XBP1 expression levels correlated negatively with the relative abundance of major MHC molecule in several cancers, including breast invasive carcinoma, cholangiocarcinoma, head and neck squamous cell carcinoma, Kidney chromophobe, brain lower grade glioma, liver hepatocellular carcinoma, prostate adenocarcinoma (Figure 5C). These results suggested that XBP1-based immunotherapy is the potentially ideal targets in most malignant cancers.

## Discussion

Although immune checkpoint inhibitors (ICI) have made breakthrough progress in cancer immunotherapy, especially in the treatment of Hodgkin’s lymphoma, melanoma and other cancers (Ribas and Wolchok, 2018), the clinical response of these inhibitors are limited in ovarian cancer (Disis et al., 2019; Matulonis et al., 2019). The targeted capability and effectiveness of immune checkpoints are based on a variety of influencing factors, including the expression or mutation in tumor and normal tissues, the contribution to cancer patient survival, as well as the relativity to antitumor immunity (Havel et al., 2019). In this study, our results showed the expression, prognosis, and relativity to antitumor immunity analyses of immune checkpoints may not be ideal therapeutic targets for ovarian cancer, and XBP1 is a potential coregulator of immune checkpoints in ovarian cancer.

Through bioinformatics analysis by web servers (GEPIA2, cBioPortal, Kaplan-Meier Plotter. TISIDB), we analyzed the expression, prognosis, and relativity to BRAC1/BRAC2 expression of all reported immune checkpoints in ovarian cancer. Summarizing the results of the comprehensive analysis, we can categorize the overall phenotypes could be categorized according to the three negative conditions. These results suggested that immune checkpoints themselves might not be ideal immunotherapy targets for ovarian cancer. However, considering the complexity of tumor immune mechanisms, growing evidence suggests that ovarian cancer likely coordinates several (but not single) immune checkpoints against tumor immune response. Moreover, individual targeting of each of immune checkpoints has variable efficacy in ovarian cancer therapy, whereas combination treatment with drugs targeting all of the checkpoints may also be restricted due to potential side effects. Thus, it is imperative to identify novel immunotherapy target in ovarian cancer.

Interestingly, the rapid progress of open high-throughput sequencing databases due to advances in whole-genome sequencing provides us with valuable information for researching potential novel immune targets for cancer therapy (Mandal et al., 2016; Luke et al., 2019). Recently, immune checkpoint inhibitor represents a breakthrough for cancer therapy, whereas immunogenic chemotherapy is also an important process in cancer immunotherapy. XBP1, an important factor mediating immunogenic chemotherapy, can exert anti-tumor immunity by regulating T cell function and dendritic cell homeostasis (Cubillos-Ruiz et al., 2015; Song et al., 2018). It may be a potential immunotherapy target.

Further bioinformatics analysis, we found that XBP1 was highly expressed in ovarian cancer, and high XBP1 expression significantly favors both overall survival and disease-free survival of patients with ovarian cancer. This also means immune checkpoint inhibitor therapy combined with chemotherapy in XBP1-upregulated cancers might be a more appropriate therapeutic strategy for ovarian cancer and provide patients with an extra survival benefit. Moreover, XBP1 was further observed to be closely associated with anti-tumor immunity in ovarian cancer, including multiple immune effector molecules and T-cell signatures. Importantly, XBP1 alteration is significantly related to multiple immune checkpoints at a genomic level, which strongly suggests a possible co-contribution to immune surveillance and evasion of ovarian cancer. Thus, upregulating XBP1 rather than targeting immune checkpoints represents a potentially more efficient approach for ovarian cancer therapy.

At the same time, there were some limitations in our study. On the one hand, it is the first report describing XBP1 to be the genomic coupler of immune checkpoints, but all the data analyzed in our study were obtained from different online databases, which might cause background heterogeneity. Of note, there are several emerging reports about the crucial roles of genomic correlation, biomarkers, transcriptional regulation, translational modulation, and posttranslational modification in immune checkpoint blockade (Hsu et al., 2018; Zhang et al., 2018; Havel et al., 2019; Keenan et al., 2019). On the other hand, the study did not conduct experiments to verify the results obtained from bioinformatics analysis. Subsequently, Further exploration of the potentially direct interplay or indirect influence between XBP1 and immune checkpoints is required, before XBP1 becomes a widely accepted prognostic indicator and therapeutic target for both clinicians and policymakers.

In summary, we analyzed the importance of immune checkpoints in ovarian cancer by using publicly available gene expression data, and propose XBP1 as a more promising target for the therapy of ovarian cancer. Our results showed that immune checkpoint inhibitor is a poor choice for ovarian cancer patients based on clinical significance of immune checkpoints in ovarian cancer. Interestingly, based on gene expression, prognosis, and relativity to multiple immune checkpoints analyses, we found that XBP1-based therapy may show a more promising prospect for ovarian cancer immunotherapy than immune checkpoint inhibitors therapy. However, the results of this study raise an interesting new hypothesis, further biological validation is required to support our conclusion.

## Data Availability

The original contributions presented in the study are included in the article/Supplementary Material, further inquiries can be directed to the corresponding authors.

## Supplementary Material

Supplementary Figure S1 Prognostic analysis of immune checkpoints in ovarian cancer. **(A–C)** Different pathological stages analysis of PD-1 **(A)**, PD-L1 **(B)** and CTLA-4 **(C)** in ovarian cancer. Differential expression of log-scaling in different pathological stages was assessed by log2(TPM+1), and a Pr(>F) < 0.05 was recognized as statistically significant. **(D)** Correlation analysis of BRCA1 and PD-1 in ovarian cancer. **(E)** Correlation analysis of BRCA1 and PD-L1 in ovarian cancer. **(F)** Correlation analysis of BRCA1 and CTLA-4 in ovarian cancer. **(G)** Correlation analysis of BRCA2 and PD-1 in ovarian cancer. **(H)** Correlation analysis of BRCA2 and PD-L1 in ovarian cancer. **(I)** Correlation analysis of BRCA2 and CTLA-4 in ovarian cancer. GEPIA generates the pair-wise gene expression correlations of two genes, or between one gene and several signatures in ovarian cancer (TCGA tumor) using Spearman method. The detailed *p*-value and R were individually presented as indicated in each panel. The statically significant difference was considered when a *p*-value is <0.05. **(J)** Pathological Correlation of Immune Checkpoints.

Supplementary Figure S2 Expression of XBP1 in multiple human cancers and paired normal tissues. GEPIA generates box plots with jitter (size = 0.5) for comparing XBP1 expression in multiple human cancers and paired normal tissues. (TCGA tumor versus TCGA normal + GTEx normal). T, tumor samples; N, normal samples. ACC, Adrenocortical carcinoma; BLCA, Bladder Urothelial Carcinoma; BRCA, Breast invasive carcinoma; CESC, Cervical squamous cell carcinoma and endocervical adenocarcinoma; CHOL, Cholangiocarcinoma; COAD, Colon adenocarcinoma; DLBC, Lymphoid Neoplasm Diffuse Large B-cell Lymphoma; ESCA, Esophageal carcinoma; GBM, Glioblastoma multiforme; HNSC, Head and Neck squamous cell carcinoma; KICH, Kidney Chromophobe; KIRC, Kidney renal clear cell carcinoma; KIRP, Kidney renal papillary cell carcinoma; LAML, Acute Myeloid Leukemia; LGG, Brain Lower Grade Glioma; LIHC, Liver hepatocellular carcinoma; LUAD, Lung adenocarcinoma; LUSC, Lung squamous cell carcinoma; MESO, Mesothelioma; OV, Ovarian serous cystadenocarcinoma;PAAD, Pancreatic adenocarcinoma; PAAD, Pancreatic adenocarcinoma; PCPG, Pheochromocytoma and Paraganglioma; PRAD, Prostate adenocarcinoma; READ, Rectum adenocarcinoma; SARC Sarcoma; SKCM, Skin Cutaneous Melanoma; STAD, Stomach adenocarcinoma; TGCT, Testicular Germ Cell Tumors; THCA, Thyroid carcinoma; THYM, Thymoma; UCEC, Uterine Corpus Endometrial Carcinoma; UCS, Uterine Carcinosarcoma; UVM, Uveal Melanoma.
