# Supplementary figures and images for: X-box Binding Protein 1 is a Potential Immunotherapy Target in Ovarian Cancer
Source: Front Genet. 2022 Aug 4;13:818917. doi: 10.3389/fgene.2022.818917 (PMC9386150; doi:10.3389/fgene.2022.818917)

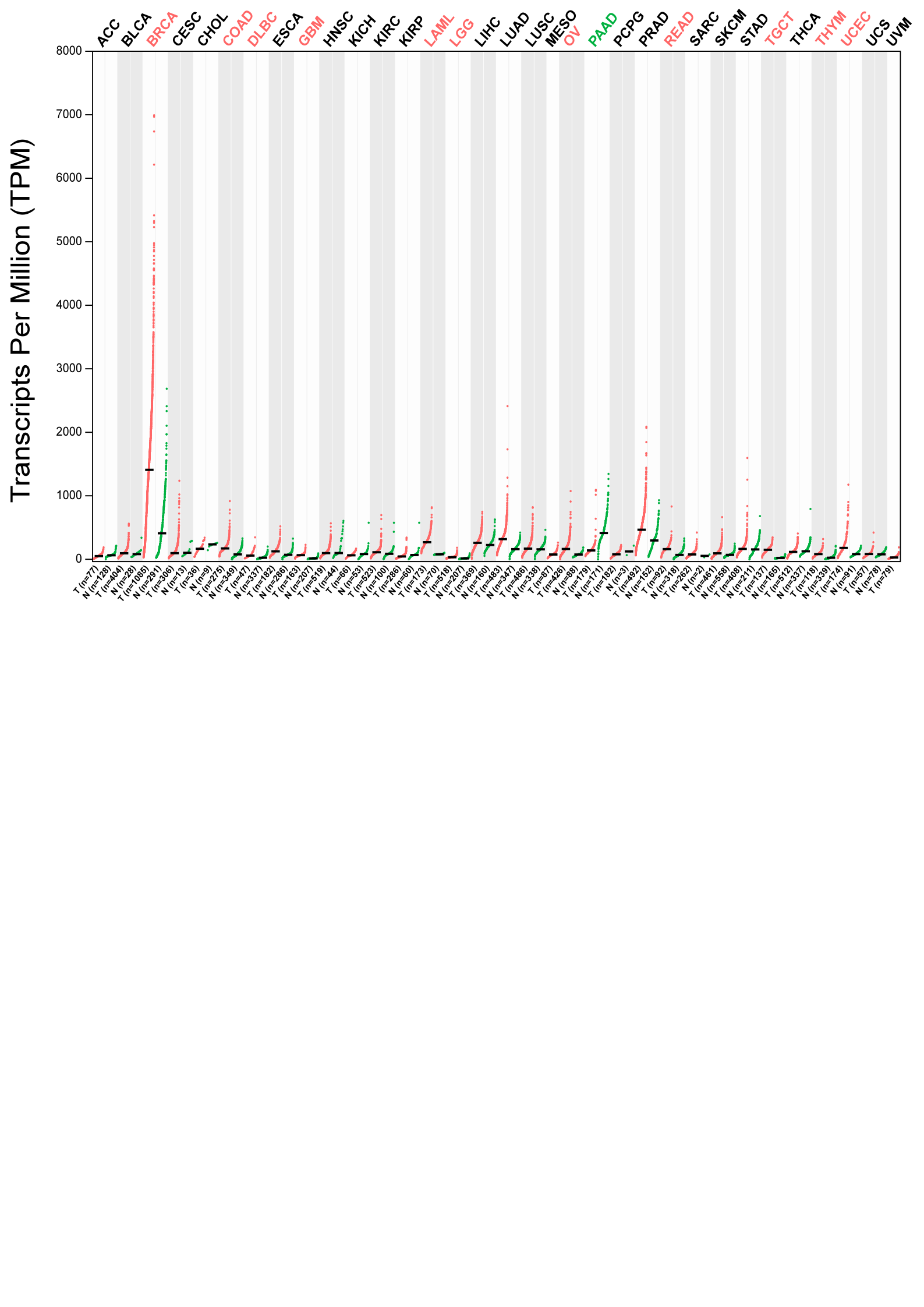

Supplement: Supplementary file 1 [file Image2.TIF]

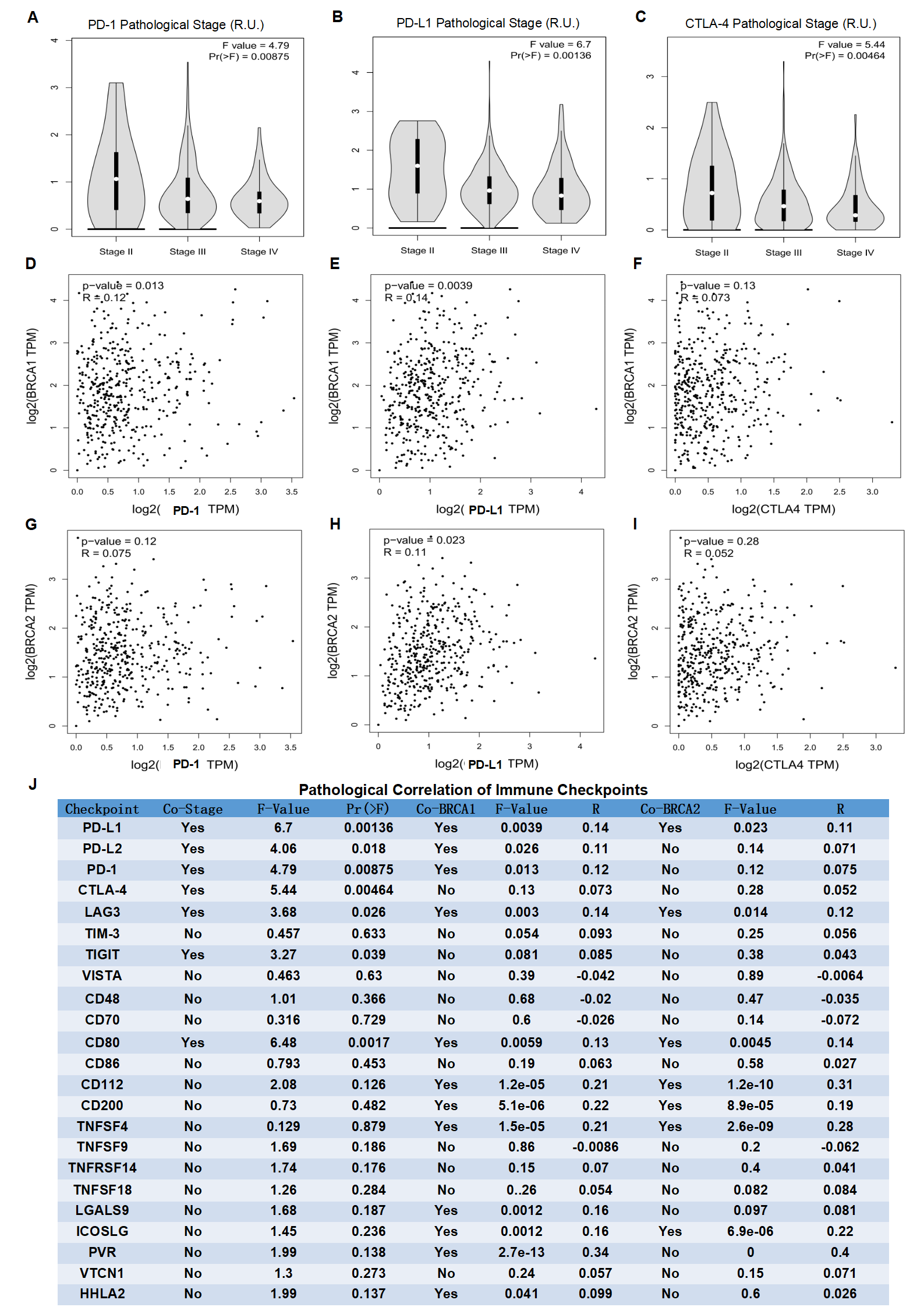

Supplement: Supplementary file 2 [file Image1.TIF]
